# Seasonal Dynamics and Nest Characterization of *Vespa orientalis* (Hymenoptera: Vespidae) in Apiaries: Insights from Bait Trap Capture Efficiency

**DOI:** 10.3390/insects17010058

**Published:** 2026-01-01

**Authors:** Sabreen G. El-Gohary, Abd El-Aziz M. A. Mohsen, Mohammed A. I. Youssif, Lamya Ahmed Alkeridis, Laila A. Al-Shuraym, Samy Sayed, Mustafa Shukry, Sherin M. M. Y. Helaly

**Affiliations:** 1 Plant Protection Department, Faculty of Agriculture, Zagazig University, Zagazig 44511, Egypt; sabreenelgohary178@gmail.com (S.G.E.-G.); abdelazizmohsen65@gmail.com (A.E.-A.M.A.M.); essmatgomaa2@gmail.com (M.A.I.Y.); sherinm82@yahoo.com (S.M.M.Y.H.); 2 Department of Biology, College of Science, Princess Nourah bint Abdulrahman University, P.O. Box 84428, Riyadh 11671, Saudi Arabia; laalkeridis@pnu.edu.sa (L.A.A.); laalshuraym@pnu.edu.sa (L.A.A.-S.); 3 Department of Economic Entomology and Pesticides, Faculty of Agriculture, Cairo University, Giza 12613, Egypt; 4 Department of Biomedical Sciences, College of Veterinary Medicine, King Faisal University, P.O. Box 400, Al-Ahsa 31982, Saudi Arabia

**Keywords:** *Vespa orientalis*, population dynamics, nest composition, bait traps

## Abstract

This study investigated the population dynamics of *Vespa orientalis* L. (Hymenoptera: Vespidae) across two consecutive seasons (2023–2024) in selected apiaries, with a focus on nest composition (eggs, larvae, and pupae) and the effectiveness of various bait traps for capturing the species. Monthly monitoring revealed the highest population peaks in October and the lowest in December. Notable inter-seasonal variations in population density were observed across the studied sites. The average number of individuals per nest varied between seasons, reflecting fluctuations in colony development and environmental factors. In 2023, the mean counts of cells, eggs, larvae, and pupae per nest were 30.14, 18.77, 13.33, and 20.88, respectively, while in 2024, they were 10.55, 14.81, 18.02, and 30.43. Among the tested attractants, grape juice proved the most effective, capturing an average of 511.67 hornets, followed by black honey (422.33 hornets), whereas the capturing trap caught only 5 hornets. These findings provide insights into the seasonal activity and reproductive status of *V. orientalis* and support the development of environmentally friendly capture strategies.

## Simple Summary

This study examined the population changes of the oriental hornet (*Vespa orientalis*) in bee yards during two successive seasons (2023–2024) in three locations in Egypt. The number of hornets was highest in October and lowest in December, with noticeable differences between years and locations. We also studied the contents of hornet nests, including eggs, larvae, and pupae, which varied between the two seasons. Among the different food baits tested, grape juice attracted the most hornets, followed by black honey, while the capturing trap caught very few. These results help us to understand the seasonal behavior of *V. orientalis* and can assist in developing safer and more effective ways to capture it around apiaries.

## 1. Introduction

The oriental hornet, *Vespa orientalis* L. (Hymenoptera: Vespidae), is native to the countries of the Mediterranean basin and the Near East [1]. It typically constructs its nests in underground cavities or within the earthen walls of old buildings [2]. After emerging from hibernation in spring, a fertilized queen searches for a suitable and protected site to establish a new colony [3]. The primary nest, which is called the embryo nest, is built by the queen only [4]. The colony undergoes rapid expansion during the summer months, reaching populations of several thousand active individuals under favorable conditions [5]. The main role of the queen is ovipositing into the comb cells, one egg per comb cell, and continuing to build additional comb cells until the brood comb contains 20–30 cells [6]. After about a month, the initial workers provided food for the developing brood and cleaned the nest [7]. At the end of the active season, from September until December, decrease the worker population and increase the number of drones. Once the enclosed queens accumulate in their bodies a sufficient supply of storage materials, they commence their wedding flight [8]. Once the enclosed queens accumulate sufficient storage materials in their bodies, they commence their nuptial flight [9]. After mating, the drones die, while the fertilized queens return to the nest and remain there until the onset of the rains, after which they leave in search of dark and dry sites to hibernate until the following spring [6]. *Vespa orientalis* L. is considered one of the most significant pests of honeybee colonies, causing severe damage in apiaries [10]. It frequently invades weak colonies, capturing larvae, pupae, pollen, and worker bees, which are then carried back to the hornet nest to feed its brood [11]. In addition to attacking honeybees, *V. orientalis* preys on various insects belonging to the orders Coleoptera, Diptera, Hemiptera, Hymenoptera, and Lepidoptera in terrestrial ecosystems [12]. Furthermore, vespid species are known to attack many fruit trees, causing damage to fruits such as grapes, figs, and dates in Egypt [13,14]. *Vespa orientalis* L. poses a threat to beekeeping, as observations have reported that a single hornet may kill dozens of honeybees per hour to feed its brood [15,16]. This study aimed to examine the seasonal abundance of this insect in both the apiary and nests. Additionally, a capture study was conducted to monitor *V. orientalis* using bait traps under field conditions.

## 2. Materials and Methods

### 2.1. Apiaries Locations

**Figure 1:**
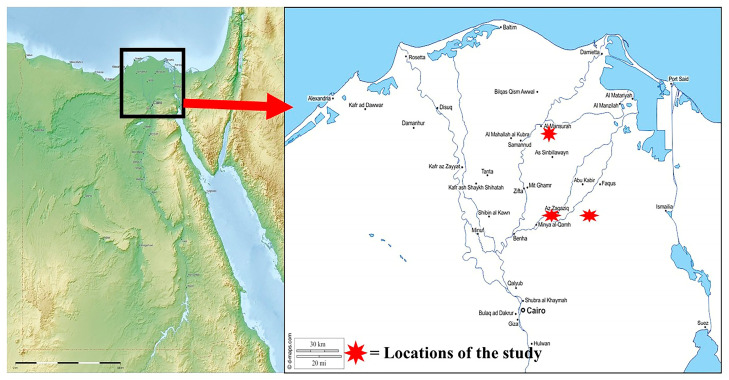
Location of the three apiaries in Dakahlia (Meet-Ghamr) and Sharkia Governorates (Bani Amir and El-Moullak), Egypt, where the seasonal abundance of *Vespa orientalis* L. was monitored during 2023–2024.

The experiment was conducted during two successive years (2023 and 2024) in three private apiaries located in Dakahlia and Sharkia Governorates, Egypt (Figure 1). The first apiary was situated in the Mansoura district of Dakahlia Governorate, while the second and third apiaries were located in the Zagazig and Abu Hammad districts of Sharkia Governorate, respectively. Each apiary consisted of 40 Langstroth hives containing healthy *Apis mellifera* L. colonies managed under standard beekeeping practices. The hives were arranged in four parallel rows, with approximately 1.5 m spacing between hives and 3 m between rows, and were placed under partial shade to reduce heat stress. The surrounding environment consisted of mixed crops, including citrus, clover, and date palms, providing natural foraging resources. The experiment started in January 2023 and continued until December 2024 to cover all seasonal activity periods of *V. orientalis*.

### 2.2. Traps

Two types of traps were used to monitor and capture *Vespa orientalis* populations, including wooden traps and bottle traps.

#### 2.2.1. Wooden Traps

**Figure 2:**
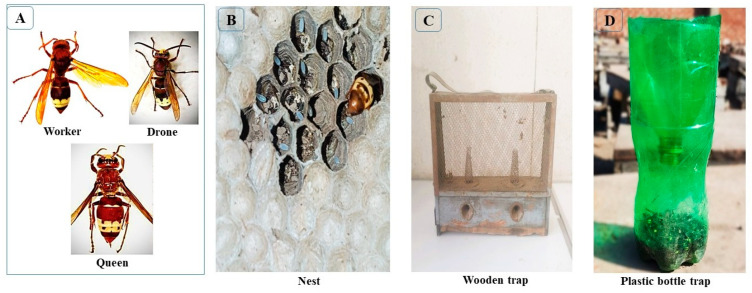
(**A**) Comparative view of the queen, worker, and drone of *V. orientalis* L. collected during the study; (**B**) *Vespa orientalis* nest collected near apiaries; (**C**) wooden trap; (**D**) plastic bottle trap.

Wooden traps (33.5 cm × 10 cm) with two sides of wire mesh and two internal wire funnels, which allow hornets to enter but prevent their exit, were used to assess the population density of *V. orientalis* (Figure 2C). Each trap had a designated compartment for bait, consisting of 100 g wheat flour, 20 mL clover honey, 10 g dry yeast, and 50 mL fermented sugar solution (total 180 g). The traps were placed randomly among the hives in each apiary, hung approximately 1.5 m above the ground in shaded areas to avoid direct sunlight, and positioned so as not to obstruct bee activity. Traps were installed at the start of the experiment in January 2023 (winter) and remained in use throughout all seasons until December 2024. Each trap was left in place for two weeks, after which the captured hornets (queens, workers, and drones) were counted, the bait was replaced, and the traps were reinstalled in the same positions. This cycle was repeated throughout the study period. Population density was determined based on the total number of hornets captured per trap. Air temperature and relative humidity in each apiary were measured using a digital thermo-hygrometer (HTC-1) placed 1.5 m above ground in a shaded location. Measurements were taken twice daily at 9:00 a.m. and 3:00 p.m., and the mean values were used to represent seasonal conditions.

#### 2.2.2. Plastic Bottle Traps

Plastic bottle traps (2 L) were prepared according to Abdullaev et al. [15] and Al-Heyari et al. [17]. Each bottle was cut 10 cm below the top, and the upper part was inverted to form a funnel that permitted hornets to enter but prevented their exit. The traps were filled with a mixture of the tested bait, including grape juice, black honey, dry yeast, tuna, cinnamon oil, vinegar, and cod liver oil, and a 1:1 (*w*/*v*) sugar solution, which served as both an attractant and a killing medium. Hornets feeding on the bait slipped into the solution and drowned.

### 2.3. Evaluation of Some Bait Effectiveness for Capturing V. orientalis Hornets in Apiaries

The baits mentioned above were tested for their attractiveness to *V. orientalis*. Each bait (20 g or mL) was added to 500 mL of the sugar solution, while the sugar solution alone was used as the control. Plastic bottle traps were distributed randomly among hives in the apiary (40 colonies), hung at approximately 1.5 m above ground level, and checked weekly to count dead hornets, replace the bait, and maintain consistent trapping conditions. Each treatment was replicated three times. Air temperature and relative humidity in each apiary were measured twice daily using a digital thermo-hygrometer (HTC-1), and mean values were used to represent seasonal conditions.

### 2.4. Vespa Orientalis Nests

Nests of *Vespa orientalis* were collected through active searches in areas surrounding the apiaries. A cotton-wool plug soaked in diethyl ether was gently inserted into the nest tunnel to immobilize the adults. *V. orientalis* nests are typically found in cracks or cavities of old mud buildings near drains and canals. The collected nests were carefully placed in polyethylene bags and transported to the laboratory. Each nest consisted of a single comb attached to the substrate and enclosed by a paper envelope, which is characteristic of *V. orientalis* nest architecture (Figure 2B). For each nest, adult and immature stages (eggs, larvae, and pupae) were counted and weighed. Nest dimensions (length, width, and height) and total nest weight were also recorded. A total of 34 nests were collected during the study period.

### 2.5. Statistical Analysis

All data underwent one-way ANOVA and post hoc Tukey’s multiple comparisons to assess the variation among the different parameters in the experimental groups. Before conducting the analysis, tests for normality (Shapiro–Wilk test) and homogeneity of variances (Levene’s test) were performed. No transformations were applied to the data, as the results of the normality and homogeneity tests indicated that the data were suitable for ANOVA without the need for transformations. All statistical analyses were conducted utilizing SPSS version 23 (IBM, Chicago, IL, USA). A *p* value of <0.05 was deemed statistically significant.

## 3. Results

### 3.1. Seasonal Abundance of Vespa orientalis L. At Three Apiaries During Two Successive Seasons, 2023 and 2024

**Figure 3:**
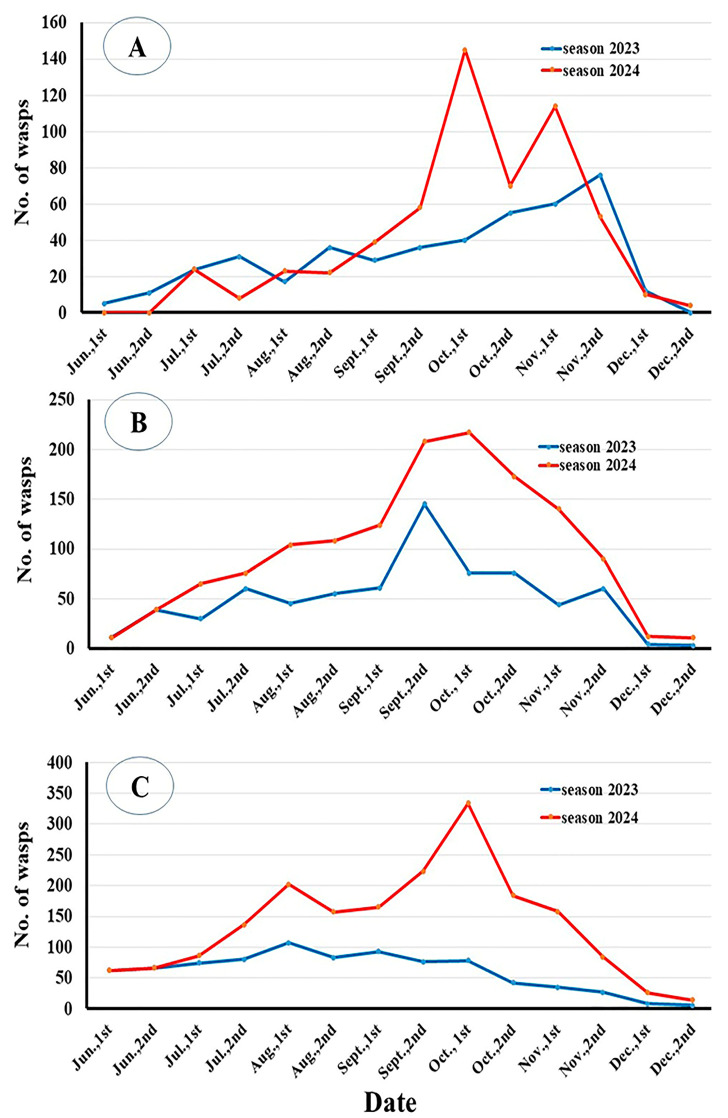
Numbers of *V. orientalis* during two seasons (2023–2024) at Meet-Ghamr (**A**), Bani Amir (**B**), and El-Moullak (**C**) apiaries.

The seasonal abundance of *V. orientalis* across the Meet-Ghamr, Bani Amir, and El-Moullak apiaries during the 2023 and 2024 seasons demonstrated notable fluctuations in the numbers of queens, drones, workers, and total hornets. At the Meet-Ghamr apiary, the total number of *V. orientalis* in 2023 and 2024 was recorded at Meet-Ghamr as 135 and 236 individuals for queens, 46 and 21 individuals for drones, and 251 and 286 individuals for workers; at Bani Amir, 426 and 239 individuals for queens, 32 and 29 individuals for drones, and 251 and 379 individuals for workers; and at El-Moullak apiaries, 535 and 391 individuals for queens, 49 and 44 individuals for drones, and 252 and 620 individuals for workers (Figure 3). The highest numbers of hornets recorded were 76 and 145 individuals in November 2023 and October 2024, respectively, at the Meet-Ghamr apiary. At the Bani Amir apiary, the peak numbers were 145 and 141 individuals in September 2023 and October 2024, respectively. Meanwhile, at the El-Moullak apiary, the highest counts were 107 individuals in August 2023 and 256 individuals in October 2024 (Tables S1–S3).

Temperature (T) and relative humidity (RH%) exhibited significant seasonal variation at all three apiaries. At the Meet-Ghamr apiary, temperatures ranged from 13.1 to 35.4 °C, while RH% fluctuated between 33.4 and 62.0%. Bani Amir apiary recorded temperatures between 11.3 and 35.8 °C, with RH% ranging from 40.4 to 74.1%. The El-Moullak apiary recorded the temperature range from 13.3 to 36.1 °C, and RH% varied between 48.7 and 72.1%. The correlation analysis between the total number of *V. orientalis* hornets and environmental factors, including T and RH%, revealed distinct patterns across the three apiaries in 2023 and 2024. At the Meet-Ghamr apiary, a positive correlation was observed between total hornet numbers and temperature, with r values of 0.42 in 2023 and 0.35 in 2024, indicating a moderate association that slightly weakened over time (Table S1). In contrast, the correlation between total hornet numbers and RH% was positive in 2023 (r = 0.40) but showed a marked decline in 2024 (r = 0.13), suggesting a diminishing influence of RH% on hornet abundance over the two years. At the Bani Amir apiary, a stronger positive correlation was found between total hornet numbers and temperature, with r values of 0.62 in 2023 and 0.50 in 2024, demonstrating a significant association between temperature and hornet population dynamics in both years. However, the correlation between total hornets and RH% remained weak, with r values of 0.12 in 2023 and 0.13 in 2024, indicating that RH% had minimal impact on hornet populations at this location (Table S2). The El-Moullak apiary displayed a notably strong positive correlation between total hornet numbers and temperature in 2023 (r = 0.83), which decreased to a moderate correlation in 2024 (r = 0.39), suggesting that temperature initially played a significant role, but its influence weakened over time. The correlation between total hornets and RH% at El-Moullak was negligible, with r values of −0.01 in 2023 and 0.03 in 2024, indicating that RH% had little to no effect on hornet population dynamics at this apiary (Table S3).

### 3.2. Efficiency of Some Bait Traps in Trapping Vespa orientalis

Table 1 showed that the efficiency of seven bait traps (Sugar solution+ grape juice, Sugar solution + black honey, Sugar solution + yeast, Sugar solution+ tuna, Sugar solution + cinnamon, Sugar solution+ vinegar, Sugar solution + cod liver oil, and Sugar solution as a control in a private apiary during the period extended from August to December 2024. The mean number of *Vespa orientalis* captured every month was 26.78, 54.16, 71.66, 27.58, and 3.37, respectively. The highest monthly number of captured hornets was 71.66 hornets in October. The highest trapped hornets were 511.67 for grape juice, followed by 422.33 for black honey, and the lowest number was 5 hornets for the control.

### 3.3. Seasonal Changes in Nest Dimensions and Brood Composition of Vespa orientalis in 2023 and 2024

Nests of *V. orientalis* were collected and examined monthly from September to December during two successive seasons (2023 and 2024). Each nest was measured for length, width, height, and weight, and the number of cells, eggs, larvae, and pupae was recorded.

The total number of nests collected during the two seasons was 16 in 2023 and 18 in 2024. As shown in Table 2, the mean nest length, width, height, and weight were 9.59, 11.02, 2.50, and 19.44, respectively, in 2023, while in 2024 they were 9.30, 14.20, 2.06, and 41.27, respectively. The mean numbers of cells, eggs, larvae, and pupae per nest were 30.14, 18.77, 13.33, and 20.88, respectively, in 2023, compared to 10.55, 14.81, 18.02, and 30.43, respectively, in 2024.

As presented in Table 3, the highest mean number of eggs (22.25) and larvae (25.50) was recorded in November 2023, while the highest number of pupae occurred in September 2023. Similarly, in the 2024 season, November recorded the greatest mean for eggs, larvae, and pupae.

## 4. Discussions

The present study evaluated the seasonal population dynamics of *V. orientalis* in three apiaries and examined the characteristics of collected nests over two consecutive seasons (2023–2024). In both years, the hornet population exhibited a distinct seasonal trend, with the maximum total count recorded during October and the minimum in December. This seasonal pattern suggests that *V. orientalis* activity is strongly influenced by temperature, consistent with the species’ known thermophilic nature and preference for warm environments [18,19]. The number of nests collected was 16 in 2023 and 18 in 2024, indicating stable establishment and nesting behavior in the studied locations.

The increase in population during late summer and early autumn coincides with the period of high foraging activity, colony expansion, and peak brood rearing. These findings are in agreement with those of Gomaa and Abd El-Wahab [20], who reported the highest numbers of *V. orientalis* during October, followed by September and November, before the population declined by late December. Similarly, Fouad et al. [21] observed that workers began appearing in July and peaked in October, followed by a gradual decrease toward winter. Mahfouz and Abd Al-Fattah [22] observed that few individuals of *V. orientalis* in the North Sinai region (Egypt) start to appear from March till May; then, a low population was observed in June, followed by a gradual increase from July up to the end of October. The relatively low activity recorded in December in the current study reflects the onset of cold weather, which suppresses hornet foraging and colony maintenance. Similar seasonal declines were documented by Beggs [23], who noted that *Vespa* activity decreased markedly in winter. This pattern mirrors the life cycle of other *Vespa* species, such as *V. crabro* and *V. velutina*, which also show peak worker abundance in late summer, followed by rapid population decline as colonies collapse before overwintering [24,25,26,27].

Regarding nesting behavior, most of the collected nests were composed of a single comb, although a few larger nests, particularly from November, showed the initiation of a second comb. Nest dimensions and weight varied between seasons, which may be attributed to differences in resource availability and colony maturity. The mean number of cells, eggs, larvae, and pupae also differed between years, possibly reflecting environmental factors such as temperature, food supply, and colony age. Similar variations were reported for *Vespa velutina* and *V. crabro*, where colony growth and brood composition were influenced by environmental conditions and prey abundance [28,29].

From an applied perspective, the evaluation of different bait traps demonstrated that grape juice was the most effective attractant, capturing an average of 511.67 hornets, followed by black honey (422.33 hornets), while the control trapped only five individuals. These results highlight the importance of using attractants with strong olfactory cues and fermentable sugars to enhance trapping efficiency. Comparable findings were reported by Ghania and Abd El-Aziem [30], who found that sugar syrup and honey baits recorded the highest captures in October and November. Similarly, Abd El-Kareim et al. [31] and Augul et al. [32] demonstrated that fermented honey and *Cupressus sempervirens* oil can effectively attract and reduce *V. orientalis* populations. Moreover, sugar syrup or honey is used as bait for cached queens, and the bait consists of fermented honey with little powder yeast and grape syrup for cached workers [10]. In other *Vespa* species, attractant performance also varies seasonally and by bait type. For instance, Al Heyari et al. [33] found sardine bait highly attractive to *V. orientalis* within the first 24 h, while Hassanein et al. [34] reported that pollen substitute baits were most successful. Abdullah et al. [35] found the average number of hornets trapped was 84.8, 98.1, 79.4, and 79.2 by taste traps with pear juice, date palm juice, grape juice, and control (the sugar solution), respectively. The variation among studies could be due to local environmental factors, differences in colony nutritional requirements, or the chemical composition of the bait materials. The preference for grape juice in the current study may be explained by its high sugar content and fermentation aroma, which mimic natural fruit volatiles attractive to hornets. Ecologically, understanding the population peaks and nesting behavior of *V. orientalis* is essential for timing capture measures effectively. The results indicate that trapping efforts should be intensified from late August through October, when colonies are most active and worker numbers are at their peak. Early intervention during this period can significantly reduce predation pressure on honeybee colonies and limit nest establishment in apiaries [36].

Overall, the findings contribute valuable insight into the population ecology of *V. orientalis* and provide a scientific basis for developing sustainable management strategies. Future studies should focus on identifying the chemical composition of effective attractants, exploring environmentally safe repellents, and integrating bait-trapping methods with other capture techniques to protect honeybee colonies from hornet attacks.

## 5. Conclusions

The present study provides valuable information on the population dynamics, nesting behavior, and capture of *V. orientalis* in apiaries over two consecutive seasons (2023–2024). The findings revealed that hornet populations peak during October and decline markedly in December, indicating a strong seasonal pattern influenced by climatic conditions and resource availability. Variations in the number of cells, eggs, larvae, and pupae per nest reflect differences in colony development between seasons. Furthermore, the efficiency of various bait traps in capturing hornets was assessed. Grape juice and black honey proved to be highly effective attractants, capturing the highest number of hornets. Overall, this study contributes to a better understanding of the biology, ecology, and potential management strategies for *V. orientalis*, providing useful information for developing integrated management approaches to protect honeybee colonies and mitigate hornet infestations in apiaries.

## Figures and Tables

**Table 1:** The efficiency of the bait type in plastic bottle traps on the captured *Vespa orientalis* at El-Moullak locality, Sharkia Governorate, Egypt, during August–December 2024.

Trapping Agents	Mean Number of *Vespa orientalis* Captured per Month					Total
	August	September	October	November	December	
Sugar solution + grape juice	81.33 ^a^	142.33 ^a^	180.33 ^b^	91 ^b^	16.66 ^a^	511.67 ^a^
Sugar solution + black honey	40 ^c^	96 ^b^	183.33 ^a^	97.33 ^a^	5.66 ^b^	422.33 ^b^
Sugar solution + yeast	46.66 ^b^	62 ^d^	84.33 ^c^	15 ^c^	1.33 ^d^	209 ^d^
Sugar solution + tuna	24.33 ^d^	91 ^c^	83.33 ^c^	16 ^c^	3.33 ^c^	218 ^c^
Sugar solution + cinnamon	13 ^e^	17 ^e^	17 ^d^	4.66 ^d^	0	51.67 ^e^
Sugar solution + vinegar	8.66 ^f^	12.66 ^f^	9 ^f^	1.66 ^e^	0	32 ^f^
Sugar solution + cod liver oil	0	10.66 ^f^	13 ^e^	5 ^f^	0	28.67 ^g^
Sugar solution (control)	0.33 ^g^	1.66 ^g^	3 ^g^	0	0	5 ^h^
Total	214.31	433.31	573.32	220.65	26.98	
Mean	26.78	54.16	71.66	27.58	3.37	
T (C°)	29.7	29.3	26	19.8	17.2	
RH%	55.8	59.7	60.2	62.1	59.7	
F ratio	96.63	19.03	33.85	190.85	45.82	

Means in each column followed by the same letter(s) are not significantly different at 5% level of probability according to Tukey’s multiple range tests.

**Table 2:** Nest census of *Vespa orientalis* during its seasonal activity, a period extending from September to December 2023.

Date	No. of Nest	Nest Measurements				No. of Cells	No. of Eggs	No. of Larvae	Weight of Larva	No. of Pupa	Weight of Pupa
		Length (cm)	Width (cm)	Height (cm)	Weight (g)						
September 2023	1	6 ± 0.6	8 ± 0.25	3 ± 0.04	18.66 ± 0.14	36	21	16	0.41 ± 0.01	29	0.66 ± 0.01
October 2023	2	5.3 ± 0.11	4.1 ± 0.94	1 ± 0.08	3.46 ± 0.06	31	23	6	0.86 ± 0.36	12	0.53 ± 0.13
		2.8 ± 0.17	3.2 ± 0.33	1.9 ± 0.11	0.44 ± 0.011	28	12	3	0.66 ± 0.41	14	0.61 ± 0.08
	Mean	4.05	3.65	1.45	1.95	29.5 ^a^	17.5 ^b^	4.5 ^c^	0.76 ^a^	13 ^b^	0.57 ^a^
November 2023	4	3 ± 0.81	3 ± 0.04	1.5 ± 0.17	0.86 ± 0.10	32	12	9	0.52 ± 0.15	17	0.39 ± 0.02
		1.6 ± 0.23	1.3 ± 0.09	0.3 ± 0.23	0.65 ± 0.07	0	6	0	0.42 ± 0.06	11	0.47 ± 0.09
		24 ± 0.81	23 ± 0.13	3 ± 0.14	86.4 ± 1.12	36	27	66	0.54 ± 0.08	24	0.67 ± 0.13
		27 ± 0.19	24 ± 0.18	6 ± 0.34	89.2 ± 2.14	31	44	27	0.42 ± 0.03	22	0.61 ± 0.11
	Mean	13.9	12.82	4.05	44.27	24.75 ^b^	22.25 ^a^	25.50 ^a^	0.47 ^b^	18.2 ^a^	0.53 ^b^
December 2023	9	7.9 ± 0.55	11 ± 0.25	1.5 ± 0.16	11.94 ± 0.02	9	43	5	0.91 ± 0.11	18	0.59 ± 0.09
		13 ± 0.14	20.5 ± 0.16	1.5 ± 0.08	3.46 ± 0.21	20	37	16	0.86 ± 0.09	40	0.53 ± 0.01
		13 ± 0.55	25 ± 0.08	1.8 ± 0.02	66.43 ± 1.18	18	33	28	0.54 ± 0.04	9	0.61 ± 0.04
		9 ± 0.94	9.5 ± 0.04	1.9 ± 0.19	7.67 ± 0.13	19	0	2	0.41 ± 0.03	8	0.64 ± 0.11
		11 ± 0.54	7.5 ± 0.13	2 ± 0.08	8.11 ± 0.06	42	11	7	0.55 ± 0.07	3	0.54 ± 0.13
		5.5 ± 1.02	10 ± 0.18	1.1 ± 0.13	3.95 ± 0.05	56	0	5	0.52 ± 0.03	21	0.52 ± 0.22
		6.8 ± 0.87	8 ± 0.22	1 ± 0.27	7.12 ± 0.07	25	0	3	0.42 ± 0.08	14	0.61 ± 0.29
		5.7 ± 0.61	7.9 ± 0.14	0.8 ± 0.03	3.45 ± 0.11	35	5	0	0	0	0
		6.5 ± 0.49	6 ± 0.29	2 ± 0.29	3.94 ± 0.14	49	0	0	0	0	0
	Mean	14.41	19.61	1.51	12.89	30.33 ^a^	14.33 ^c^	7.33 ^b^	0.46 ^b^	12.55 ^b^	0.44 ^c^
General mean		9.59	11.02	2.50	19.44	30.14	18.77	13.33	0.52	20.88	0.55

Means in each column followed by the same letter(s) are not significantly different at 5% level of probability according to Tukey’s multiple range tests. The values are shown in means ± SE. The means within the same row carrying different superscripts are significant at *p* < 0.05.

**Table 3:** Nest census of *Vespa orientalis* during its seasonal activity, a period extending from September to December 2024.

Date	No. of Nests	Nest Measurements				No. of Cell	No. of Eggs	No. of Larvae	Weight of Larva	No. of Pupa	Weight of Pupa
		Length (cm)	Width (cm)	Height (cm)	Weight (g)						
September 2024	3	7 ± 0.25	8.7 ± 0.49	3 ± 0.01	30.03 ± 0.21	9	23	5	0.41 ± 0.06	7	0.61 ± 0.01
		8 ± 0.61	13 ± 0.11	2.9 ± 0.04	52.78 ± 0.01	8	16	9	0.45 ± 0.04	0	0.52 ± 0.03
		9.5 ± 0.87	11 ± 0.09	2.5 ± 0.08	40.5 ± 0.84	13	15	18	0.45 ± 0.03	19	0.58 ± 0.04
	Mean	8.16 ^c^	10.9 ^c^	2.80 ^a^	41.10 ^b^	10 ^b^	18 ^b^	10.66 ^c^	0.43 ^a^	13 ^c^	0.57 ^a^
October 2024	2	10.5 ± 0.36	17 ± 0.05	1.5 ± 0.41	20.07 ± 0.13	12	11	4	0.4 ± 0.01	0	0.53 ± 0.11
		12 ± 0.87	12.5 ± 0.56	2 ± 0.11	58.7 ± 0.18	9	23	36	0.49 ± 0.03	15	0.63 ± 0.09
	Mean	11.25 ^a^	14.75 ^b^	1.75 ^b^	39.38 ^b^	10.5 ^b^	17 ^b^	20 ^b^	0.44 ^a^	7.50 ^d^	0.58 ^a^
November 2024	7	13 ± 0.63	14 ± 0.57	1.5 ± 0.36	32.07 ± 0.05	12	61	0	0.57 ± 0.05	84	0.69 ± 0.08
		9.5 ± 1.02	19 ± 0.64	2 ± 0.54	76.1 ± 0.17	11	35	43	0.51 ± 0.03	98	0.53 ± 0.03
		15.5 ± 1.18	20 ± 0.14	1 ± 0.12	12.13 ± 0.96	8	10	22	0.48 ± 0.02	77	0.61 ± 0.01
		7.5 ± 0.24	22.5 ± 0.07	2 ± 0.24	59.9 ± 0.36	9	11	16	0.49 ± 0.01	81	0.55 ± 0.02
		7 ± 0.87	35.5 ± 0.16	2 ± 0.98	91.07 ± 0.14	10	19	23	0.41 ± 0.06	70	0.43 ± 0.04
		12 ± 0.55	22 ± 0.01	1.4 ± 0.74	76.13 ± 0.18	9	23	82	0.45 ± 0.01	65	0.56 ± 0.07
		12 ± 0.14	13 ± 0.19	2.5 ± 0.46	83 ± 0.55	9	5	41	0.45 ± 0.08	26	0.71 ± 0.05
	Mean	10.93 ^b^	20.85 ^a^	1.77 ^b^	61.48 ^a^	9.71 ^c^	23.42 ^a^	32.42 ^a^	0.48 ^a^	71.57 ^a^	0.58 ^a^
December 2024	6	7.5 ± 0.97	10 ± 0.22	2 ± 0.98	20.13 ± 2.14	12	1	4	0.49 ± 0.02	57	0.61 ± 0.01
		6.2 ± 0.55	14 ± 0.36	2.4 ± 0.54	30.04 ± 1.36	16	4	34	0.46 ± 0.06	6	0.61 ± 0.01
		10 ± 0.41	11 ± 0.47	2 ± 0.12	27.54 ± 0.85	9	0	3	0.51 ± 0.01	23	0.6 ± 0.01
		8 ± 0.77	14.5 ± 0.63	2.3 ± 0.36	53.53 ± 0.61	16	0	4	0.68 ± 0.03	9	0.7 ± 0.01
		4.5 ± 0.54	6 ± 0.05	1.5 ± 0.17	3.58 ± 0.58	10	0	5	0.41 ± 0.04	26	0.6 ± 0.01
		5 ± 0.71	6.5 ± 0.17	1.4 ± 0.64	3.9 ± 1.02	9	0	4	0.42 ± 0.01	57	0.72 ± 0.02
	Mean	6.86 ^d^	10.33 ^c^	1.93 ^b^	23.12 ^c^	12 ^a^	0.83 ^c^	9 ^c^	0.49 a	29.6 ^b^	0.64 ^a^
General mean		9.3	14.20	2.06	41.27	10.55	14.81	18.02	0.46	30.43	0.59

Means in each column followed by the same letter(s) are not significantly different at 5% level of probability according to Tukey’s multiple range tests. The values are shown in means ± SE. The means within the same row carrying different superscripts are significant at *p* < 0.05.

## Data Availability

The original contributions presented in this study are included in the article/Supplementary Material. Further inquiries can be directed to the corresponding authors.

## Supplementary Materials

The following supporting information can be downloaded at: https://www.mdpi.com/article/10.3390/insects17010058/s1, Table S1: Seasonal abundance of *Vespa orientalis* L. at Meet-Ghamr apiary during the two seasons 2023 and 2024. Table S2: Seasonal abundance of *Vespa orientalis* L. at Bani Amir apiary during the two seasons 2023 and 2024. Table S3: Seasonal abundance of *Vespa orientalis* L. at El-Moullak apiary during the two seasons 2023 and 2024.
