# Supplementary material for: Seasonal Dynamics and Nest Characterization of Vespa orientalis (Hymenoptera: Vespidae) in Apiaries: Insights from Bait Trap Capture Efficiency
Source: Insects. 2026 Jan 1;17(1):58. doi: 10.3390/insects17010058 (PMC12842075; doi:10.3390/insects17010058)
Supplement: Supplementary file 1 [file insects-17-00058-s001.zip › insects-4053352-supplementary.pdf]

**Table S1.** Seasonal abundance of *Vespa orientalis* L. at Meet-Ghamr apiary during the two seasons 2023 and 2024.

| Date of samples | Numbers of | | | | | | Total no. of wasps | | °c | | RH% | |
|---|---|---|---|---|---|---|---|---|---|---|---|---|
| | Queens | | Drones | | Workers | | | | | | | |
| | 2023 | 2024 | 2023 | 2024 | 2023 | 2024 | 2023 | 2024 | 2023 | 2024 | 2023 | 2024 |
| Jan., 1st | 0 | 0 | 0 | 0 | 0 | 0 | 0 | 0 | 14.3 | 14.0 | 47.2 | 64.7 |
| 2nd | 0 | 0 | 0 | 0 | 0 | 0 | 0 | 0 | 12.4 | 16.6 | 37.0 | 65.3 |
| Feb., 1st | 0 | 0 | 0 | 0 | 0 | 0 | 0 | 0 | 17.0 | 14.4 | 33.4 | 54.5 |
| 2nd | 0 | 0 | 0 | 0 | 0 | 0 | 0 | 0 | 13.1 | 15.4 | 61.5 | 52.1 |
| Mar., 1st | 0 | 0 | 0 | 0 | 0 | 0 | 0 | 0 | 14.8 | 15.5 | 48.5 | 54.4 |
| 2nd | 0 | 0 | 0 | 0 | 0 | 0 | 0 | 0 | 18.5 | 19.5 | 47.2 | 74.7 |
| Apr., 1st | 0 | 0 | 0 | 0 | 0 | 0 | 0 | 0 | 16.9 | 19.3 | 49.9 | 53.7 |
| 2nd | 0 | 0 | 0 | 0 | 0 | 0 | 0 | 0 | 18.0 | 21.8 | 72.4 | 47.4 |
| May., 1st | 0 | 0 | 0 | 0 | 0 | 0 | 0 | 0 | 30.0 | 25.3 | 49.1 | 36.5 |
| 2nd | 0 | 0 | 0 | 0 | 0 | 0 | 0 | 0 | 29.8 | 26.6 | 45.7 | 54.0 |
| Jun., 1st | 0 | 0 | 0 | 0 | 5 | 0 | 5 | 0 | 29.7 | 28.7 | 41.2 | 31.0 |
| 2nd | 0 | 0 | 0 | 0 | 11 | 0 | 11 | 0 | 29.1 | 26.6 | 54.0 | 43.3 |
| Jul., 1st | 13 | 24 | 0 | 0 | 11 | 0 | 24 | 24 | 30.0 | 28.4 | 59.9 | 46.1 |
| 2nd | 15 | 8 | 0 | 0 | 16 | 0 | 31 | 8 | 29.9 | 28.9 | 54.6 | 62.0 |
| Aug., 1st | 9 | 15 | 0 | 0 | 8 | 8 | 17 | 23 | 30.0 | 30.9 | 59.8 | 49.2 |
| 2nd | 24 | 11 | 0 | 0 | 12 | 11 | 36 | 22 | 35.4 | 30.0 | 26.1 | 58.8 |
| Sept., 1st | 17 | 8 | 0 | 0 | 12 | 31 | 29 | 39 | 29.4 | 30.8 | 59.2 | 51.4 |
| 2nd | 22 | 9 | 0 | 0 | 14 | 49 | 36 | 58 | 28.2 | 28.4 | 54.8 | 59.1 |
| Oct., 1st | 10 | 51 | 7 | 1 | 23 | 93 | 40 | 145 | 26.0 | 28.2 | 59.1 | 57.4 |
| 2nd | 13 | 7 | 12 | 5 | 30 | 58 | 55 | 70 | 27.1 | 26.1 | 70.2 | 66.4 |
| Nov., 1st | 8 | 67 | 5 | 3 | 47 | 20 | 60 | 114 | 22.2 | 23.2 | 59.2 | 58.1 |
| 2nd | 4 | 31 | 21 | 7 | 51 | 15 | 76 | 53 | 22.8 | 19.8 | 62.3 | 59.6 |
| Dec., 1st | 0 | 5 | 1 | 2 | 11 | 3 | 12 | 10 | 19.9 | 17.6 | 69.6 | 41.2 |
| 2nd | 0 | 0 | 0 | 3 | 0 | 1 | 0 | 4 | 15.3 | 17.0 | 45.1 | 53.6 |
| Total | 135 | 236 | 46 | 21 | 251 | 286 | 432 | 570 | | | | |
| Mean | 11.25 | 19.66 | 3.8 | 1.75 | 20.91 | 23.83 | 18 | 23.75 | | | | |

| r value: | 2023 | 2024 |
|---|---|---|
| - Between total no. of wasps and T | 0.42 | 0.35 |
| - Between total no. of wasps and RH% | 0.40 | 0.13 |

**Table S2.** Seasonal abundance of *Vespa orientalis* L. at Bani Amir apiary during the two seasons 2023 and 2024.

| Date of samples | Numbers of | | | | | | Total no. of wasps | | °C | | RH% | |
|---|---|---|---|---|---|---|---|---|---|---|---|---|
| | Queens | | Drones | | Workers | | | | | | | |
| | 2023 | 2024 | 2023 | 2024 | 2023 | 2024 | 2023 | 2024 | 2023 | 2024 | 2023 | 2024 |
| Jan., 1st | 0 | 0 | 0 | 0 | 0 | 0 | 0 | 0 | 13.1 | 11.8 | 58.1 | 52.7 |
| 2nd | 0 | 0 | 0 | 0 | 0 | 0 | 0 | 0 | 11.3 | 13.8 | 58.1 | 74.1 |
| Feb., 1st | 0 | 0 | 0 | 0 | 0 | 0 | 0 | 0 | 15.7 | 14.2 | 61.3 | 65.2 |
| 2nd | 0 | 0 | 0 | 0 | 0 | 0 | 0 | 0 | 14.8 | 15.4 | 45.9 | 69.1 |
| Mar., 1st | 0 | 0 | 0 | 0 | 0 | 0 | 0 | 0 | 16.6 | 16.3 | 41.0 | 56.2 |
| 2nd | 0 | 0 | 0 | 0 | 0 | 0 | 0 | 0 | 17.0 | 15.3 | 54.2 | 64.5 |
| Apr., 1st | 0 | 0 | 0 | 0 | 0 | 0 | 0 | 0 | 19.4 | 22.5 | 46.7 | 63.5 |
| 2nd | 0 | 0 | 0 | 0 | 0 | 0 | 0 | 0 | 18.4 | 19.6 | 57.3 | 61.3 |
| May., 1st | 0 | 0 | 0 | 0 | 0 | 0 | 0 | 0 | 23.1 | 23.7 | 52.0 | 50.8 |
| 2nd | 0 | 0 | 0 | 0 | 0 | 0 | 0 | 0 | 26.3 | 31.7 | 43.9 | 42.9 |
| Jun., 1st | 6 | 0 | 0 | 0 | 5 | 0 | 11 | 0 | 30.1 | 26.0 | 43.5 | 40.4 |
| 2nd | 18 | 0 | 0 | 0 | 21 | 0 | 39 | 0 | 29.0 | 32.5 | 53.1 | 49.6 |
| Jul., 1st | 11 | 35 | 0 | 0 | 19 | 0 | 30 | 35 | 30.6 | 30.5 | 46.1 | 50.7 |
| 2nd | 24 | 16 | 0 | 0 | 36 | 0 | 60 | 16 | 35.8 | 27.9 | 51.9 | 53.8 |
| Aug., 1st | 25 | 39 | 0 | 0 | 20 | 21 | 45 | 59 | 31.3 | 31.1 | 53.9 | 53.3 |
| 2nd | 26 | 4 | 1 | 0 | 28 | 49 | 55 | 53 | 30.3 | 29.9 | 58.1 | 53.9 |
| Sept., 1st | 41 | 12 | 7 | 0 | 13 | 51 | 61 | 63 | 28.2 | 29.3 | 55.7 | 62.6 |
| 2nd | 93 | 7 | 4 | 0 | 48 | 56 | 145 | 63 | 28.1 | 29.8 | 60.3 | 62.2 |
| Oct., 1st | 51 | 39 | 1 | 1 | 24 | 101 | 76 | 141 | 28.0 | 26.6 | 49.2 | 56.1 |
| 2nd | 56 | 22 | 10 | 2 | 10 | 73 | 76 | 97 | 26.0 | 24.5 | 48.0 | 56.3 |
| Nov., 1st | 34 | 45 | 0 | 15 | 10 | 36 | 44 | 96 | 24.7 | 22.0 | 62.0 | 57.1 |
| 2nd | 40 | 12 | 9 | 10 | 11 | 8 | 60 | 30 | 21.6 | 19.5 | 64.4 | 60.1 |
| Dec., 1st | 1 | 6 | 0 | 1 | 3 | 1 | 4 | 8 | 18.1 | 18.6 | 56.2 | 60.4 |
| 2nd | 0 | 2 | 0 | 5 | 3 | 1 | 3 | 8 | 15.7 | 17.6 | 70.0 | 72.1 |
| Total | 426 | 239 | 32 | 29 | 251 | 397 | 706 | 669 | | | | |
| Mean | 35.5 | 19.91 | 2.66 | 2.41 | 20.91 | 33.08 | 29.54 | 27.87 | | | | |

| r value: | 2023 | 2024 |
|---|---|---|
| - Between total no. of wasps and T | 0.62 | 0.50 |
| - Between total no. of wasps and RH% | 0.12 | 0.13 |

**Table S3.** Seasonal abundance of *Vespa orientalis* L. at El-Moullak apiary during the two seasons 2023 and 2024.

| Date of samples | Numbers of | | | | | | Total no. of wasps | | °C | | RH% | |
|---|---|---|---|---|---|---|---|---|---|---|---|---|
| | Queens | | Drones | | Workers | | | | | | | |
| | 2023 | 2024 | 2023 | 2024 | 2023 | 2024 | 2023 | 2024 | 2023 | 2024 | 2023 | 2024 |
| Jan., 1st | 0 | 0 | 0 | 0 | 0 | 0 | 0 | 0 | 13.9 | 11.7 | 65.4 | 59.9 |
| 2nd | 0 | 0 | 0 | 0 | 0 | 0 | 0 | 0 | 13.3 | 13.9 | 62.0 | 69.6 |
| Feb., 1st | 0 | 0 | 0 | 0 | 0 | 0 | 0 | 0 | 18.2 | 15.3 | 50.6 | 67.4 |
| 2nd | 0 | 0 | 0 | 0 | 0 | 0 | 0 | 0 | 13.6 | 14.0 | 57.4 | 57.1 |
| Mar., 1st | 0 | 0 | 0 | 0 | 0 | 0 | 0 | 0 | 16.6 | 29.1 | 54.2 | 57.0 |
| 2nd | 0 | 0 | 0 | 0 | 0 | 0 | 0 | 0 | 17.9 | 24.8 | 57.4 | 51.2 |
| Apr., 1st | 0 | 0 | 0 | 0 | 0 | 0 | 0 | 0 | 28.3 | 30.1 | 53.7 | 50.8 |
| 2nd | 0 | 0 | 0 | 0 | 0 | 0 | 0 | 0 | 28.5 | 30.3 | 50.3 | 53.5 |
| May., 1st | 0 | 0 | 0 | 0 | 0 | 0 | 0 | 0 | 26.2 | 35.8 | 56.7 | 50.1 |
| 2nd | 0 | 0 | 0 | 0 | 0 | 0 | 0 | 0 | 28.6 | 32.1 | 50.7 | 54.7 |
| Jun., 1st | 38 | 0 | 0 | 0 | 24 | 0 | 62 | 0 | 29.1 | 35.1 | 50.8 | 50.7 |
| 2nd | 52 | 0 | 0 | 0 | 55 | 0 | 66 | 0 | 30.9 | 36.1 | 48.7 | 55.4 |
| Jul., 1st | 44 | 12 | 0 | 0 | 30 | 0 | 74 | 12 | 29.7 | 30.1 | 50.6 | 50.8 |
| 2nd | 46 | 56 | 0 | 0 | 34 | 0 | 80 | 56 | 30.8 | 38.2 | 50.0 | 50.9 |
| Aug., 1st | 34 | 69 | 0 | 0 | 32 | 26 | 107 | 95 | 31.3 | 38.8 | 50.3 | 50.7 |
| 2nd | 72 | 41 | 0 | 0 | 21 | 33 | 83 | 74 | 29.4 | 35.8 | 50.3 | 59.6 |
| Sept., 1st | 50 | 5 | 5 | 0 | 21 | 67 | 93 | 72 | 28.9 | 30.2 | 53.1 | 58.8 |
| 2nd | 61 | 20 | 7 | 0 | 15 | 127 | 76 | 147 | 27.5 | 30.6 | 58.1 | 53.7 |
| Oct., 1st | 27 | 65 | 3 | 5 | 12 | 186 | 78 | 256 | 29.5 | 30.9 | 56.1 | 58.4 |
| 2nd | 65 | 13 | 9 | 6 | 4 | 104 | 42 | 142 | 25.9 | 27.3 | 51.5 | 53.5 |
| Nov., 1st | 24 | 82 | 7 | 9 | 4 | 51 | 35 | 123 | 24.2 | 22.0 | 53.2 | 56.7 |
| 2nd | 11 | 17 | 16 | 21 | 0 | 19 | 27 | 57 | 21.9 | 20.9 | 58.1 | 62.0 |
| Dec., 1st | 6 | 11 | 2 | 0 | 0 | 7 | 8 | 18 | 18.5 | 18.2 | 57.0 | 68.6 |
| 2nd | 5 | 0 | 0 | 3 | 0 | 6 | 5 | 9 | 15.8 | 17.0 | 69.4 | 72.1 |
| Total | 535 | 391 | 49 | 44 | 252 | 620 | 836 | 1061 | | | | |
| Mean | 44.58 | 32.58 | 4.08 | 3.66 | 21 | 51.66 | 34.83 | 44.20 | | | | |

| r value: | 2023 | 2024 |
|---|---|---|
| - Between total no. of wasps and T | 0.83 | 0.39 |
| - Between total no. of wasps and RH% | - 0.01 | 0.03 |
